# Sustainable development goals applied to digital pathology and artificial intelligence applications in low- to middle-income countries

**DOI:** 10.3389/fmed.2023.1146075

**Published:** 2023-05-15

**Authors:** Sumi Piya, Jochen K. Lennerz

**Affiliations:** ^1^Nepal Medical College and Teaching Hospital (NMCTH), Kathmandu, Nepal; ^2^Nepal Cancer Hospital and Research Center, Lalitpur, Nepal; ^3^Department of Pathology, Center for Integrated Diagnostics, Massachusetts General Hospital, Harvard Medical School, Boston, MA, United States

**Keywords:** machine learning, innovation, resource constraints, low- to middle-income countries, sustainable development goals

## Abstract

Digital Pathology (DP) and Artificial Intelligence (AI) can be useful in low- and middle-income countries; however, many challenges exist. The United Nations developed *sustainable development goals* that aim to overcome some of these challenges. The *sustainable development goals* have not been applied to DP/AI applications in low- to middle income countries. We established a framework to align the 17 *sustainable development goals* with a 27-indicator *list* for low- and middle-income countries (World Bank/WHO) and a list of 21 *essential elements* for DP/AI. After categorization into three domains (*human factors*, IT/*electronics, and materials + reagents*), we permutated these layers into 153 concatenated statements for prioritization on a four-tiered scale. The two authors tested the subjective ranking framework and endpoints included ranked sum scores and visualization across the three layers. The authors assigned 364 points with 1.1–1.3 points per statement. We noted the prioritization of human factors (43%) at the *indicator* layer whereas IT/electronic (36%) and human factors (35%) scored highest at the *essential elements* layer. The authors considered goal 9 (industry, innovation, and infrastructure; average points 2.33; sum 42), goal 4 (quality education; 2.17; 39), and goal 8 (decent work and economic growth; 2.11; 38) most relevant; intra-/inter-rater variability assessment after a 3-month-washout period confirmed these findings. The established framework allows individual stakeholders to capture the relative importance of sustainable development goals for overcoming limitations to a specific problem. The framework can be used to raise awareness and help identify synergies between large-scale global objectives and solutions in resource-limited settings.

## Introduction

The *sustainable development goals* are a set of 17 global goals set by the United Nations in 2015 and recently adopted in the 2030 agenda (1–5). The goals provide a “shared blueprint for peace and prosperity for people and the planet, now and into the future.” The goals cover a wide range of issues, including poverty, hunger, health, education, climate change, gender equality, water, sanitation, energy, the environment, and social justice (5, 6). The goals are intended to be universal, meaning they apply to all countries, regardless of their level of development (1, 4, 5, 7–11). They are also integrated, meaning they recognize that the various goals are interconnected and that progress in one area can have positive impacts on other areas. The goals are based on the principle of leaving no one behind, and they aim to ensure that all people can live healthy, productive lives, and to enjoy the benefits of economic and social progress (6, 9–11). The goals are ambitious and provide goals for governments, businesses, civil society, and individuals to work together to build a more sustainable and equitable world (9, 11, 12). Many organizations have followed and contribute to these goals. To our knowledge these goals have not been applied to Digital Pathology and or Artificial Intelligence (AI) applications in low- to middle income countries.

Digital pathology refers to the use of computer technology to analyze and manage information from digital images of tissue samples (13). These digitized images are typically obtained by scanning microscope slides that have been stained with a special dye to highlight the features of the tissue. Digital pathology allows pathologists to diagnose diseases more easily and accurately, as well as to share images and information with other medical professionals. It can also be used to develop algorithms and other tools to aid in the analysis of tissue samples (13–16). Digital pathology has the potential to be useful in low- and middle-income countries in several ways. One of the main advantages is that it allows pathologists to share images and information more easily with other medical professionals, even if they are in different parts of the world (17–19). Data sharing can be particularly beneficial in countries where there may be a shortage of qualified pathologists. Digital pathology can also make it easier to diagnose diseases, particularly those that require a high level of expertise to identify (17–19). This can help to improve the accuracy and reliability of diagnoses, which can lead to better patient outcomes. Additionally, digital pathology can help to reduce the cost of tissue analysis (20, 21), which can be a significant barrier to access in low- and middle-income countries. Overall, the use of digital pathology in low- and middle-income countries has the potential to improve the quality and accessibility of pathology services, which can ultimately benefit patients in these countries (13–21). The specific barriers of implementation are highly context dependent and how the specific requirements of digital pathology relate to the *sustainable development goals* remains currently uncharted.

Artificial intelligence (AI) has the potential to enhance diagnostic pathology in several ways (11, 13, 22). AI algorithms can be trained to recognize patterns in tissue samples that may be difficult for human pathologists to detect. This can help to improve the accuracy and reliability of diagnoses and can also aid in the identification of rare or unusual diseases. In addition, AI can be used to analyze large amounts of data from tissue samples, which can help to identify trends and patterns that may not be apparent to human pathologists. This can be especially useful in the early detection and treatment of diseases, as well as in the development of new diagnostic techniques and treatments. Simply put, the use of AI in diagnostic pathology has the potential to improve the accuracy and reliability of diagnoses, and to provide pathologists with new tools and insights to aid in their work. Similarly, AI can be used in a variety of ways to improve the lives of people low- and middle-income countries. For example, AI can be used to develop diagnostic tools that are more affordable and accessible for people in low- and middle-income countries (23–25). AI has the potential to help improve the accuracy and reliability of diagnoses and can also make it easier for people to access the healthcare they need. Overall, the use of AI in low- and middle-income countries has the potential to improve healthcare systems and thereby the lives of millions of people; however, the relevance of the sustainable development goals in this context of AI in digital pathology has not been assessed.

Here we prioritize *sustainable development goals* by assessing their subjective relevance to digital pathology and AI applications in low- to middle-income countries. By prioritizing sustainable developmental goals as applied to digital pathology and AI applications in low- and middle-income countries, we hope to raise awareness for these meaningful goals and provide an approach to identify synergies between *sustainable development goals* and innovations in diagnostic pathology.

## Methods

### Study design

The project site was Massachusetts General Hospital/Harvard Medical School, Department of Pathology, Center for Integrated Diagnostics, in Boston, MA. The project was conducted as a composition and alignment of three data sources followed by subjective scoring and analysis. The idea for this project emerged from a presentation during an internship in November 2022 where the authors reviewed quality management systems including relevant standards. The *International Organization for Standardization* (ISO) follows and contributes to the sustainable development goals.^1^ The project was conducted as part of a quality improvement initiative and does not require formal review (institutional checklist).

### Data sources

The project utilized three data components: (a) the 17 *sustainable development goals* (Supplementary Table 1),^2^ (b) an *indicator list* for low- and middle-income countries (Supplementary Table 2); and (c) a list of *essential elements* for digital pathology and AI (Supplementary Table 3). The list of *indicators* of low- and middle-income countries was a combination of the list of World Development Indicators (WDI) created by the World Bank and the Global diffusion of eHealth by the WHO (26, 27) (Supplementary Table 2). The *essential elements* relevant for the implementation of digital pathology/AI were selected from prior publications (13–21). Each item in the *essential elements* and the *indicator* list was assigned to one of three domains: human factors, IT/electronics, or materials + reagents (Supplementary Tables 2, 3).

### Alignment, scoring, and analysis

For alignment, the authors utilized the three data sources. The authors permutated all three indicator domains, with each of the seventeen *sustainable development goals,* and all three essential element domains (3 × 17 × 3 = 153). The resulting 153 concatenations represent the alignment. For scoring, each concatenation was compiled into a sentence that followed the following format: “When considering the specific low- and middle-income country conditions/limitations imposed by <*indicator domain*>, how important do you think is addressing the <sustainable development goal> in helping to realize the <*essential element domain>*?” (Supplementary Table 4). The two authors tested the framework by independently ranking these statements. For ranking, the authors agreed on an ordinal, subjective, four-tiered scoring system with 0 representing “not applicable” and 1, 2, and 3, representing “low,” “medium,” and “high” relevance, respectively. Of note, we chose a four-tier scale to minimize central tendency bias. Independent (blinded) scoring was followed by combined (unblinded) analysis. To identify the most relevant sustainable development goals, we used the ranked sum across all concatenations (primary endpoint). As secondary endpoints, we used the sum and average scores per *sustainable development goal* and the absolute difference, per item and between authors (discrepancy analysis). After a three-months wash out period, the authors performed re-scoring and analyzed intra- and inter-rater variability. For visualization we used a Sankey graph (last accessed on 1/3/2023).^3^ Statistical testing was performed with Prism (Graphpad Software Inc., San Diego, CA). Statistical significance was defined as *P* < 0.05.

## Results

### Sustainable development goals

**Figure 1:**
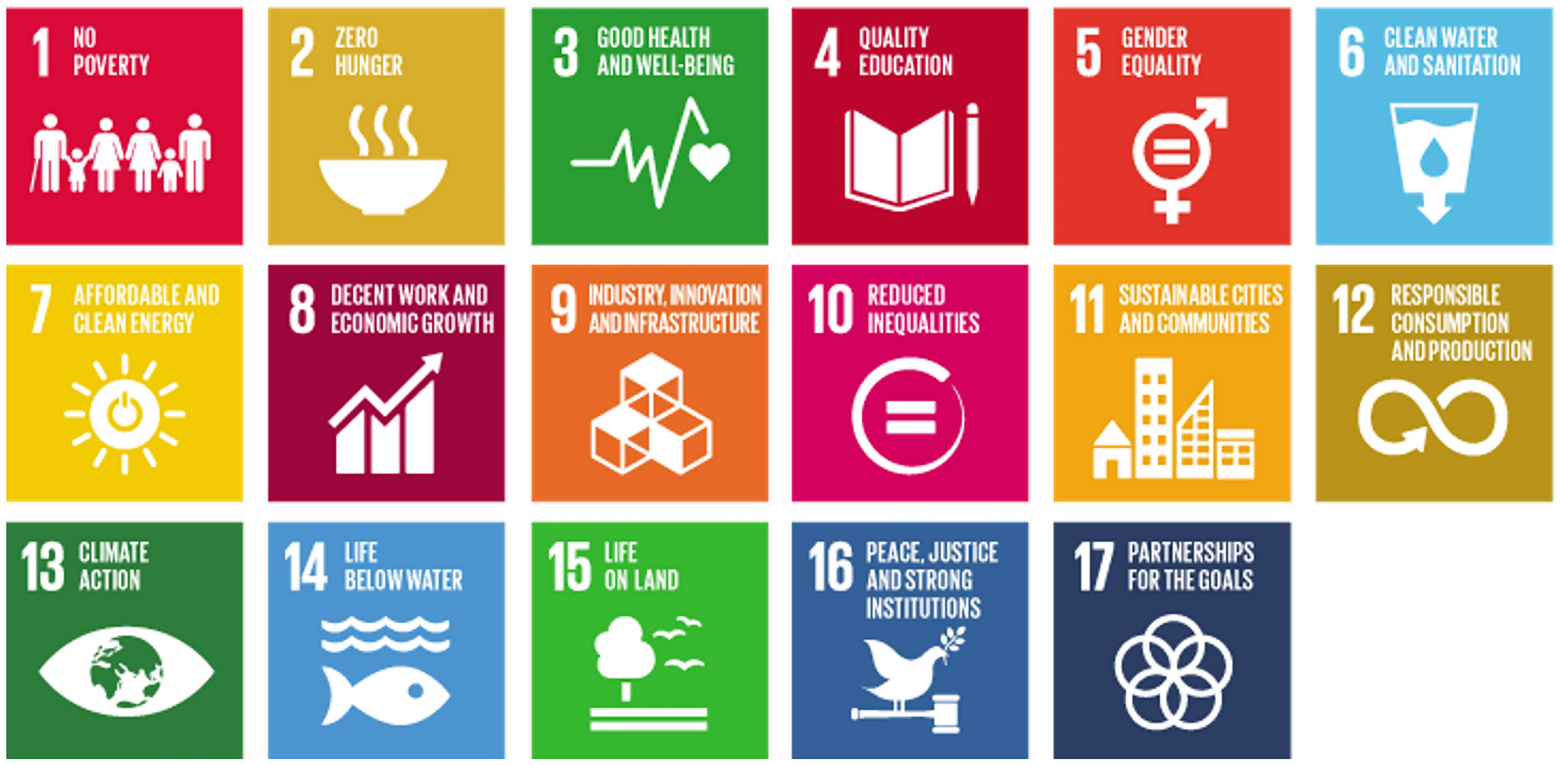
Sustainable development goals. The *Sustainable Development Goals* or Global Goals are a collection of 17 interlinked objectives designed to serve as a “*shared blueprint for peace and prosperity for people and the planet, now and into the future*.” The goals are part of the 2030 Agenda for sustained development that has been adopted by all United Nations Member States in 2015. The United Nations (Department of Economic and Social Affairs) maintains the goals and details targets, events, publications, and actions can be found at: https://sdgs.un.org/goals.

The structure of goals, targets and indicators has been published (2, 5, 28–30) and is actively disseminated for free via the United Nations’, Department of Economic and Social Affairs (Supplementary Table 1). A diagram listing the 17 *sustainable development goals* is shown in Figure 1. Many of the goals are integral part of political processes (e.g., international agreements on biodiversity, climate, or programs set by the WHO) (1–6, 12, 28–36). For example, ISO follows and contributes to the goals (16, 35) and we reviewed selected ISO projects before creating our scoring framework. Here we compiled the *sustainable development goals* (in conjunction with two independent data sources) to raise awareness and assess applicability to digital pathology and AI in low- to middle-income countries. The *sustainable development goals* formed the central layer of our analysis.

### Indicators of low- and middle-income countries

To derive a manageable list of relevant indicators for low- and middle-income countries, we extracted items from two different resources: first, the list of World Development Indicators (WDI) created by the World Bank (12, 37), and second, the document *Global diffusion of eHealth* by the WHO (26). Extraction and combination resulted in a short list of *n* = 27 indicators (Supplementary Table 2). While designed primarily as statistical features of economic activity, we used these indicators as a starting point to identify limitations for realizing digital pathology and AI in low- to middle income countries. We grouped these indicators into three domains: human factors, IT/electronics, and materials + reagents. Distribution of the 27 indicators showed that *n* = 13/27 (48%) of points were assigned to the IT/electronics domain. In contrast, *n* = 11/27 indicators were assigned to human factors (41%), and only *n* = 3/27 (11%) to the materials + reagents domain (Supplementary Table 2). The indicator list forms one adoptable layer of our analysis that captures the key limitations imposed by low-to middle income countries.

### Essential elements for digital pathology and AI

To compose the list of essential elements for the realization of digital pathology and AI, the authors reviewed several key publications (13, 17, 19–21). We focused on general requirements, laboratory and technical considerations, information technology requirements, legal and regulatory issues. After de-duplication, the authors identified *n* = 21 essential elements (Supplementary Table 3). To enable a direct comparison of the essential elements layer to the limitations layer, we grouped the *n* = 21 essential elements into the same three domains. We identified *n* = 11/21 items in the IT/electronics group (52%) whereas the human factors and materials + reagents domain contained *n* = 5 elements (24%), each. The essential elements list for digital pathology and AI forms the third layer of our applicability analysis.

### Alignment of limitations, goals, and essential elements for digital pathology and artificial intelligence

**Figure 2:**
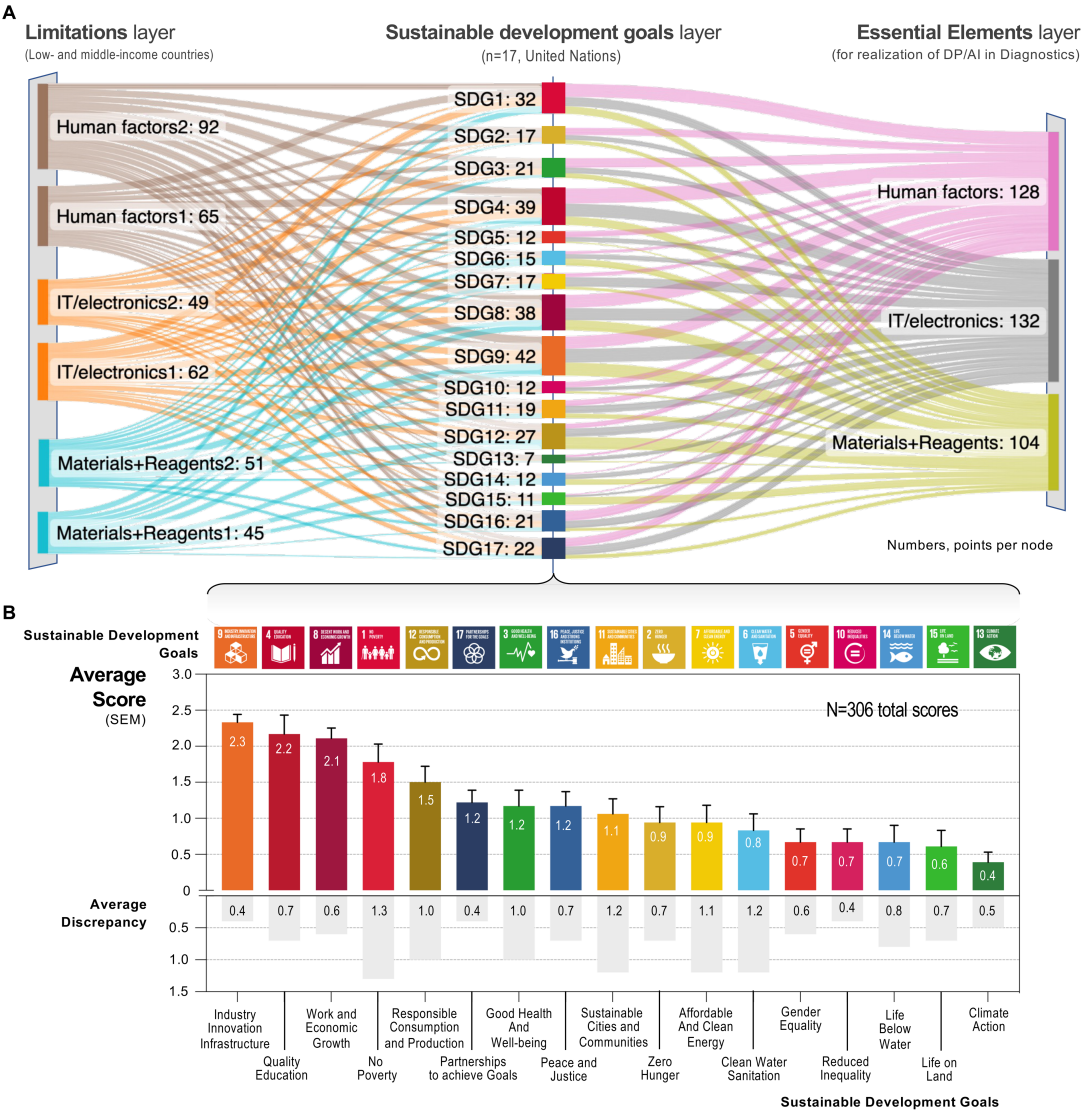
Alignment and ranking of sustainable development goals. **(A)** Sankey diagram illustrating the relationship between the three layers: limitations in low- to middle-income countries (left), the 17 *sustainable development goals* (middle), and the essential elements for realizing digital pathology/AI. The numbers indicate the total sum per node at each layer. **(B)** Average scores with standard error of the mean (error bar) by *sustainable development goals* alongside the average inter-rater discrepancy (details: Supplementary Tables 1–5). *Abbreviations:* DP, digital pathology; IT, information technology; SDG, *sustainable development goals* (numbered SDG1-SDG17); SEM, standard error of the mean.

For alignment of the three layers, we chose a point-based scoring system of single sentences that followed a concatenation format (see *Methods*). In total we assigned 364 points with an average of 1.1–1.3 points per statement. The individual points per statement are provided in Supplementary Table 4. For analysis we first combined points for each layer (Supplementary Table 5) and visualized the flow of points using a Sankey diagram (Figure 2A). At the limitations layer, the points were distributed *n* = 157 to human factors (43%), *n* = 111 to IT/electronics (30%), and *n* = 96 to materials + reagents (26%). These numbers highlight a 10% deviation from equal distribution toward human factors (range: 4–15%; Supplementary Table 5).

By ranked scores (maximum of *n* = 72 points per goal), the authors considered goal 9 (industry, innovation, and infrastructure; average points 2.33; sum 42), goal 4 (quality education; 2.17; 39), and goal 8 (decent work and economic growth; 2.11; 38) most relevant for realizing digital pathology and AI in overcoming limitations in low- to middle-income countries (Figure 2B and Supplementary Tables 4, 5). As an example, the statement (“*When considering the specific low- and middle-income country conditions/limitations imposed by IT/electronics, how important do you think is addressing goal 9: industry, innovation, and infrastructure in helping to realize the essential IT/electronic elements?*”) ranked the highest. It is noteworthy that the average discrepancies of scores for the three most relevant goals was lower than that for the other goals (0.6 vs. 0.8); however, this difference did not reach statistical significance (*P* = 0.15, Student’s *t*-test). Notably, goal 13 (climate action average points 0.39; sum 7), goal 15 (life on land; 0.61; sum 11), and goal 14 (life below water; 0.67; sum 12) received the lowest scores (Figure 2B); again, with relatively low discrepancy scores (range: 0.5–0.8).

At the *essential elements* layer, the points were distributed *n* = 128 to human factors (35%), *n* = 111 to IT/electronics (36%), and *n* = 96 to materials + reagents (29%). These findings indicate, in contrast to the limitations layer, only a 5% deviation of assigned points from equal distribution. Namely human factors and IT/electronics received more points when compared to materials + reagents (Supplementary Table 5). We point out that materials + reagents received 5–7% less points at the *limitations* and *essential elements* layer.

To assess reproducibility, the authors rescored the statements (blinded to their prior results) after a 3-months washout period. Notably, both raters gave overall lower scores. The intra-rater variability (21–27 points) was larger than the interrater variability (14–20 points). Expressed as the percent difference across all points the intra-rater variability ranged from 3.1 to 4.4% whereas the interrater variability ranged from 4.5 to 5.8%. A detailed overview of these assessments is provided in Supplementary Table 6. Importantly, despite these differences, inter- and interrater assessment in terms of ranking of goals indicated identical ranks of the first seven goals. Thus, we consider the approach robust in identifying the most relevant sustainable development goals for digital pathology and artificial intelligence applications in low- to middle-income countries.

## Discussion

Here we present and tested an alignment approach of the *sustainable development goals* to digital pathology and AI applications in low- to middle-income countries. The use of these technologies has the potential to support the achievement of several of the *sustainable development goals*, and, in reverse, pursuit of certain goals aligns well with essential components relevant to realize these innovative technologies. Raising awareness of *sustainable development goals* is key in accomplishing the underlying objectives. The presented approach, while subjective, provides a framework to align local demands with larger scale initiatives.

To our knowledge, *sustainable development goals* have not been specifically applied to digital pathology or AI applications in low- to middle-income countries. Conceptually there are two approaches to align *sustainable development goals* to a specific use case: bottom-up vs. top-down. In a bottom up (chosen here) we align the specific requirements for a use case with these large-scale goals. Akin to Sachs et al. (10), we chose this approach for two reasons: first, we interpreted the lack of relevant literature as an indicator that many pathologists might not be aware of the *sustainable development goals* and their relevance to the field—whereas, second, many pathologists are aware of the benefits of digital pathology and AI use-cases. Our approach identified goals 9 (industry innovation infrastructure), 4 (quality education), and 8 (work and economic growth) as well-aligned with the *essential elements* for digital pathology and AI. We find it interesting that these goals ranked consistently higher than sustainable development goal 3 (good health and wellbeing) (1–3, 30, 33). We considered one underlying reason that digital pathology and AI rely heavily on technologies combined with computational skills (i.e., higher education); which emphasizes the sustainability associated with improved education. Importantly, perceptions of *sustainability* often do not cover all dimensions and focus mainly on environmental aspects (2, 4, 30, 38, 39). In our rank-order goals 13–15 (climate action, life below water, life on land) were not considered as important to realize AI (11, 22, 25). Here, we noted that both authors independently considered human factors as the principal limitations (Figure 2A left layer), whereas IT/electronics and human factors are considered more important than materials + reagents at the essential elements layer (Figure 2A right layer).

Our findings are relevant for several reasons. First, we provide a concrete scoring approach that enables colleagues who are not familiar with these goals to examine their personal priorities (Supplementary Table 4). Second, from an individual investigators’ perspective, identification of relevant funding lines is an important factor for sustained innovation. The prioritization of large-scale goals, relevant to a specific question (here AI), enables identification of synergistic large-scale initiatives. For example, goal 4 (quality education) provides concrete data on improper infrastructure and contributing projects that entail virtual learning (39–42). The relevance here is, that individual laboratories can engage practically (inclusion in virtual learning sessions) as well as through resource management (e.g., identification of local or national funding initiatives) (3, 21, 38, 43). Third, in a globally interconnected world, the prioritization of digital healthcare technologies should also include low- to middle-income countries (27, 37, 44, 45). Many ambitious plans remain as statements and our approach enables a slightly more focused view. In fact, alignment of ambitious goals with practical action starts at the individual level. Finally, we acknowledge that unlocking the power of both remote diagnostics as well as AI relies heavily on numbers and diversity of cases. Thus, we consider our work a concrete starting point to explore an emergent international data market that rightfully includes low- to middle-income countries.

Limitations of our study are primarily related to the design. As intended, the prioritization presented here is clearly subjective and context dependent. Thus, we do not claim generalizability and cannot measure how useful the results are for a broader group of people or settings. However, we invite the interested reader to try the alignment for themselves by providing an interactive worksheet (Supplementary Table 4). The reader can then determine usefulness of the data –and more importantly– the approach matched to their priorities and setting(s). A clear indication for the subjectivity of our ranking is that goal 3 (good health) ranked only 7th. Of course, we presume that the use of digital pathology and AI in diagnostic pathology can help to improve the accuracy and reliability of diagnoses, which can lead to better patient outcomes (i.e., goal 3) (10, 29, 33, 39, 46); however, other goals clearly ranked higher; for example, goal 4 (quality education). Digital pathology and AI can be used to develop new educational tools and resources, which can help to improve access to education (goal 4). And the use of these technologies can help to create new job opportunities and support economic growth, which can contribute to the achievement of goal 8. While we ranked the *sustainable development goals* in this way, our aim was to prioritize *sustainable development goals* in support of a technology rather than to prioritize technologies that support *sustainable development goals*. Simply put, the chosen prioritization is a limitation that aligns the use-case to the macroscopic development goal. Another limitation is that AI has many other applications; however, we focused on AI in healthcare and specifically diagnostic pathology. Other use cases of AI include for example applications that can help farmers to increase their yields and reduce the amount of water and other resources they need (47), which can be especially beneficial in countries where resources are limited. Additionally, AI can be used to analyze large amounts of data to identify trends and patterns that can help policymakers and other decision makers to make more informed decisions (48, 49). This can be especially useful in areas such as public health, where timely and accurate information is critical to effective decision making. Finally, the authors introduce conscious and unconscious biases. For example, these biases are often based on our background, experiences, and social interactions, and they can influence how we perceive and value specific elements. Given these limitations, we recommend approaching our endpoints with caution. Despite the above-mentioned, biases our discrepancy analysis (average of 0.7 points), intra- and interrater variability assessment (Supplementary Table 6), and error modeling confirmed robust ranking of the top goals.

In summary, our study aligned *sustainable development goals* to digital pathology and AI in low- and middle-income countries. We hope that the approach will assist colleagues in identifying relevant initiatives (e.g., funding lines), support policy makers in prioritizing digital healthcare initiatives, and help to raise awareness of these ambitious goals. *Sustainable development goals* have not been previously applied to digital pathology and AI in low- to middle-income countries. The approach presented here can help identify synergies between large-scale global objectives and the potential for innovation diagnostic solutions in resource-limited settings.

## Data availability statement

The original contributions presented in the study are included in the article/Supplementary material, further inquiries can be directed to the corresponding author.

## Author contributions

All authors listed have made a substantial, direct, and intellectual contribution to the work and approved it for publication.

## Funding

This work was funded in part by the NIH (RO1 CA225655) to JL, and the content was solely the responsibility of the authors and does not necessarily represent the official views of the National Institute of Health or any other organization.

## Conflict of interest

The authors declare that the research was conducted in the absence of any commercial or financial relationships that could be construed as a potential conflict of interest.

## Publisher’s note

All claims expressed in this article are solely those of the authors and do not necessarily represent those of their affiliated organizations, or those of the publisher, the editors and the reviewers. Any product that may be evaluated in this article, or claim that may be made by its manufacturer, is not guaranteed or endorsed by the publisher.

## Footnotes

^1^https://www.iso.org/home.html

^2^https://sdgs.un.org/

^3^https://sankeymatic.com
